# Travel behaviour and Edinburgh’s Low Emission Zone: a cross-sectional survey

**DOI:** 10.3310/nihropenres.13891.2

**Published:** 2026-01-19

**Authors:** William Mueller, Fiona S. Carson, Helena Copsey, Miranda Loh

**Affiliations:** 1 Institute of Occupational Medicine (IOM), Research Ave N, EH14 4AP, Edinburgh, UK

**Keywords:** Active travel, Modal shift, LEZ, Intervention

## Abstract

**Background:**

Low Emission Zones (LEZs) aim to improve air quality and encourage sustainable travel in urban areas by issuing fines to non-compliant vehicles. This study investigated initial impacts on travel behaviour and perceptions following the enforcement of Edinburgh's LEZ from June 2024.

**Methods:**

We conducted an online cross-sectional survey focussing on commuting behaviour inside and outside the LEZ, as well as prior to and several months after enforcement. The survey targeted adults who worked or studied in Edinburgh or otherwise travelled to the LEZ area. We collected data on demographics, travel habits, and reasons for travel choices. To maximise response, we recruited participants via an online survey panel (‘panel survey’ [ *n*=351]) and social media (‘community survey’ [ *n*=260]), for which statistical analysis was stratified.

**Results:**

Following LEZ enforcement, there was a clear differentiation in transport mode usage, with more people using the car alone for their commute outside the LEZ and more people using public transport inside the LEZ. Few respondents changed whether they accessed the LEZ area. Nevertheless, comparing inside to outside commuters, 70 (44%) versus 8 (10%) in the panel survey, and 41 (24%) versus 4 (9%) in the community survey, respectively, reported some change in the frequency, duration, or mode of transport following enforcement. Results suggested a small, but statistically significant, shift towards active travel and public transport within the LEZ, with a decrease in private vehicle use. Panel survey respondents tended to agree more than community respondents with the potential positive impacts of the LEZ.

**Conclusions:**

We found evidence that active travel and public transport use increased within the first few months after the Edinburgh LEZ was enforced. Future research with objective, quantitative data should confirm these findings and assess impacts over a longer time-period.

## Plain Language Summary

This research involved an online survey of adults who worked or studied in, or travelled to, Edinburgh's Low Emission Zone (LEZ). The survey collected data on travel habits and reasons for travel choices. We also asked people’s opinions about potential impacts of the LEZ. We were interested in whether there were changes within several months after enforcement began in June 2024, which was when fees started to be charged for higher polluting cars.

The results showed differences in how people travelled inside and outside the LEZ, as well as before and after enforcement. More people used cars outside the LEZ and used public transport inside the LEZ. Following enforcement, there was a small shift towards walking, cycling, and using public transport within the LEZ.

This study showed that more people walked, cycled and used public transport in the several months following enforcement of the LEZ. Future studies should look at the LEZ and how people travel over a longer period.

## Background

Urban transportation has been linked to at least 14 pathways to health. Benefits, such as opportunities for physical activity, are relevant for active and sustainable travel, whereas negative impacts, including air pollution, stress, and road traffic injuries, relate to motorised transport ( Glazener *et al.*, 2021). According to the Scottish Health Survey ( Birtwistle *et al.*, 2022), over one third (35%) of adults did not meet the UK physical activity guidelines. As physical activity has broad physical and mental health benefits ( Anderson & Durstine, 2019), active travel (i.e., ‘travel in which the sustained physical exertion of the traveller directly contributes to their motion’ [ Cook *et al.*, 2022]) may be an important way to help meet physical activity guidance for optimal health.

Low Emission Zones (LEZs) aim to improve air quality by restricting the most polluting vehicles in certain areas (typically city centres with higher air pollutant concentrations). Fines are issued for vehicles that do not comply with emission standards ( Broster & Terzano, 2024). LEZs have been associated with reductions in air pollutants, especially particulate matter of <2.5 µm (PM _2.5_) and nitrogen dioxide (NO _2_) ( Broster & Terzano, 2024), as well as cardiovascular disease ( Chamberlain *et al.*, 2023) and paediatric respiratory admissions ( Wise, 2024). Research has associated the implementation of LEZs with an acceleration of cleaner vehicles ( Ellison *et al.*, 2013), as well as a decrease in private vehicle use and increase in public transport ( Tarriño-Ortiz *et al.*, 2022). Active travel has been linked to the London Ultra-LEZ, which at present covers all London boroughs ( TfL, 2023). Bike sharing rose by nearly 30%, notably for short and intermediate trips ( Ding *et al.*, 2023), and children attending schools within London’s Ultra-LEZ were three times more likely to switch to active travel after one year ( Xiao *et al.*, 2024). Nevertheless, LEZs may have real or perceived negative impacts, such as social exclusion ( De Vrij & Vanoutrive, 2022) or displacement of traffic elsewhere ( Moral-Carcedo, 2024).

The City of Edinburgh, UK commenced LEZ enforcement in June 2024. Our study (TRAVel behaviour & Edinburgh Low Emission Zone [TRAVEL]) involved an online survey to extend the limited evidence base on how LEZs are associated with active travel. We formulated the following two research questions to compare travel inside and outside the Edinburgh LEZ, as well as before and after enforcement:

1. How does travel behaviour differ across journeys that include and exclude the LEZ?
2. How has LEZ enforcement influenced decisions to travel to the city centre?

## Methods

### Patient and Public Involvement

A stakeholder group was established early in the TRAVEL study, which included representatives from City of Edinburgh Council, Public Health Scotland, Sustrans, NHS Lothian, Royal Town Planning Institute, Transport Scotland, and a local community member. This group provided input into the survey content and language, recruitment of potential survey respondents via social media advertising, and interpretation of results. This group will also assist with the dissemination of study findings.

***Setting.*** Edinburgh has a population of 500,000 ( CEC, 2023a) with an annual mean temperature of 13 °C and 128 days of rainfall on average ( Met Office, 2024). Edinburgh has an extensive bus service with 110 million journeys recorded in 2023 ( Lothian Buses, 2024). Bus travel is free for residents of Scotland under the age of 22 years, 60 years or older, or with a disability. Edinburgh also has a tramline and train station in the city centre.

**Figure 1.**
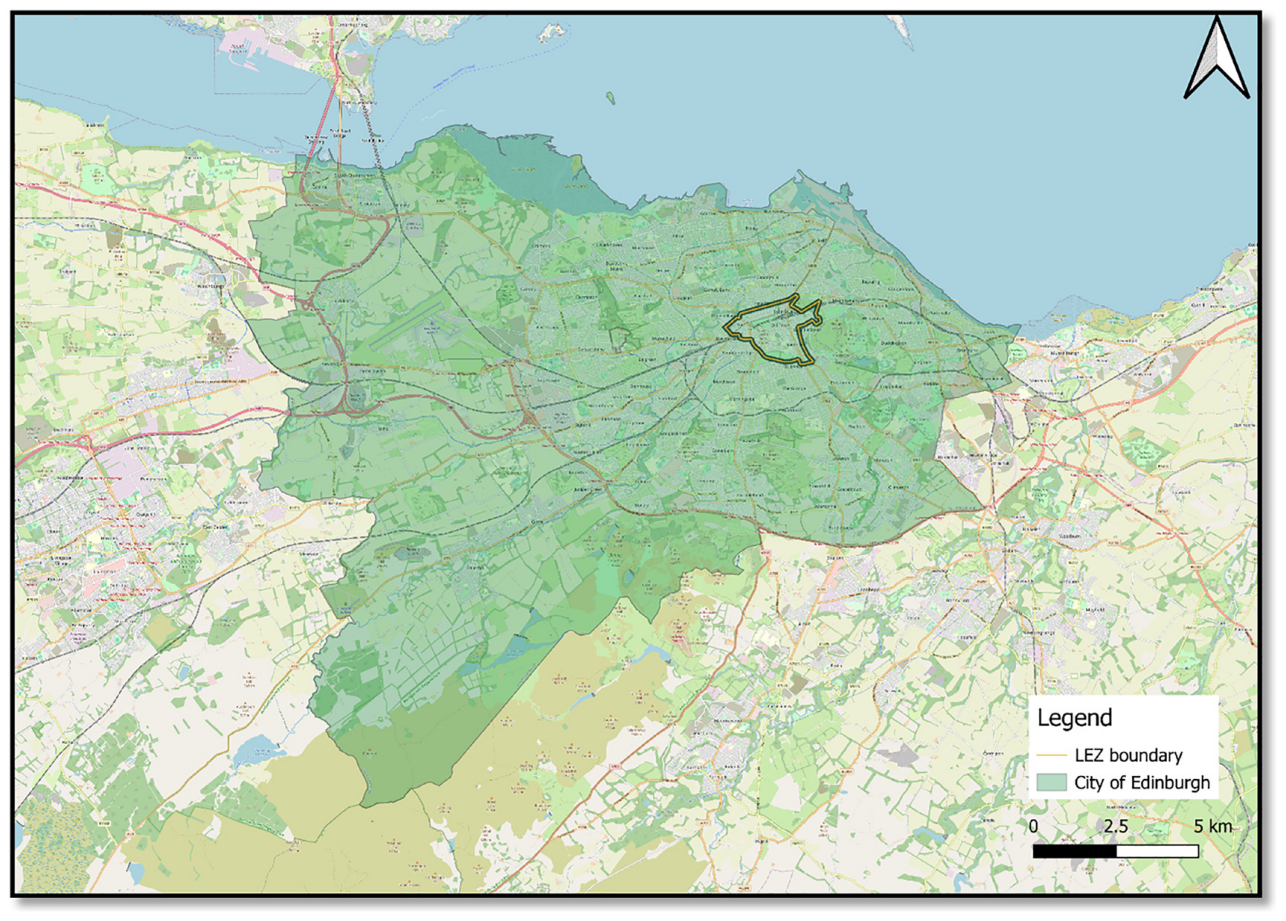
A map presenting the boundary of the Edinburgh Low Emission Zone (LEZ). Basemap from ©OpenStreetMap contributors ( www.openstreetmap.org), available under the Open Database License.

***LEZ intervention.*** The LEZ was officially implemented on 31 ^st^ May 2022 with a two-year grace period: enforcement of non-compliance fees (starting at £60 and doubling thereafter) commenced on 1 ^st^ June 2024 ( CEC, 2024b; CEC, 2024c). The LEZ allows entry of petrol cars/vans registered from 2006 with Euro 4 emission standards (Euro 6 for diesel cars/vans registered from 2015) ( CEC, 2023b). Although 95% of petrol cars in Edinburgh were estimated to have been compliant at the time of LEZ implementation, only 50% of diesel cars met the required standard ( CEC, 2023c). Funds were made available to support low-income households with the disposal of vehicles that were not LEZ-compliant, as well as vouchers for the purchase of bikes or public transport tickets ( CEC, 2024a). The LEZ is to ensure NO _2_ concentrations are below regulatory limits, to encourage more sustainable and active journeys, and to contribute to achieving net zero emissions by 2030. The LEZ covers <3% of the city with a total area of 3.1 km ^2^ ( CEC, n.d.), mainly including the city centre ( Figure 1).

***Survey design.*** To answer the two main research questions, the survey collected data on demographics, travel habits, and reasons for travel choices. To explore modal change, questions addressed travel behaviour prior to the LEZ enforcement (pre-June 2024), as well as at the time of survey submission (October–November 2024). We included, as a measure of self-reported health, personal wellbeing questions ( ONS, 2016). The main focus of the survey was the commute to work or study, with questions included for non-commute (i.e., other) travel. In summary, the questionnaire included the following content:

- Commute frequency, mode, duration (separately for inside/outside the LEZ)
- Any changes to the above (with reasons) since LEZ enforcement
- Non-commute travel inside the LEZ
- Changes in travel behaviour due to knowledge of the LEZ
- Agreement on potential impacts of the LEZ
- Age, sex, self-reported health and wellbeing

We developed the questionnaire with a professional survey company (Civica). The survey was hosted on Civica’s external survey platform, Alchemer, for which Civica have a license. The survey was piloted for clarity and ease of completion with colleagues not on the project team and was designed to be completed within 15–20 minutes. The introductory webpage that potential respondents were directed to was used as the participant information sheet. Participants provided explicit written agreement to complete the survey before progressing to the questions. The study protocol was published on Research Registry on 30 ^th^ July 2024 (UIN: researchregistry10542), and the Reading Independent Ethics Committee approved the study on 23 ^rd^ August 2024 (reference: 230824). 

***Survey recruitment.*** The target audience was adults (≥18 years old) working or studying in Edinburgh. We aimed to obtain responses from 350 individuals, using quotas to ensure representativeness in age and sex, as well as to limit respondents who did not commute (e.g., remote work/study only), but travelled to the LEZ for other reasons. The sample size was estimated using figures from the 2011 Scotland census as the most recent data available: a population size of 300,000 for the number of workers in Edinburgh, proportion of commute mode ranging from 5% (cycle) to 33% (car), margin of error of 5%, and a 95% confidence interval.

Individuals were identified, screened, and recruited through an existing panel of potential survey respondents (managed by Dynata) living in the Edinburgh area. Individuals accessed the survey through a weblink, which opened to a webpage describing the study’s purpose, content, and use of data. To maximise the number of respondents, the survey was also made available on the IOM website and was advertised by organisations represented on the stakeholder group; thus, there were two versions of the survey for ‘panel’ and ‘community’ respondents (full survey version available at https://osf.io/at8sv/). No additional payments or incentives were provided for completion of the survey; however, panel respondents do receive point incentives for each survey completed.

***Statistical analysis.*** We were interested in comparing travel behaviour of those commuting inside the LEZ to that of those whose commute did not traverse the LEZ. For the purposes of the survey, inside the LEZ was defined as a journey that passed through or included any part of the LEZ, whereas journeys outside the LEZ did not enter the LEZ at any point. The main analysis involved summary statistics of main survey questions and comparing travel behaviour responses by inside/outside LEZ commuters. Statistical analysis was undertaken to compare differences in commuting behaviour and perspectives before/after LEZ enforcement and whether travel involved the LEZ. We used several different methods depending on whether the data was categorical (chi-squared), Normal (t-test)/non-Normal (Kruskal-Wallis), paired (Wilcoxon signed rank test), or proportions (equality of proportions). The modes of transport were grouped into public transport (train, tram, and bus), active travel (bike, scooter, walk, and run), and vehicle (all other modes). Area-level deprivation (i.e., by datazone) was assigned using deciles of the Scottish Index of Multiple Deprivation (SIMD) matched to postcode. Respondents had the opportunity to include as free text any further comments on the LEZ. These remarks were broadly classified into positive (i.e., in support of the LEZ), negative (i.e., opposed to the LEZ), or neutral (i.e., neither in support nor opposed).

Analysis was stratified for the panel and community respondents, given the different recruitment methods. Only fully completed surveys were included in the data analysis, which was performed using R (R Core Team, Vienna, Austria).

***Carbon emissions.*** As an additional analysis, given the LEZ will contribute to Edinburgh’s 2030 Climate Strategy (CEC, 2021), we analysed the total commuting carbon emissions before and after enforcement for single modes of those commuting inside the LEZ. Emission estimates were based on reported mode, duration, and weekly frequency. As we did not have data on distance travelled, we estimated speed by mode via published sources, including the Physical Activity compendium ( Hermann *et al.*, 2024).

The following speeds were used for the single modes reported in the panel survey results:

- **Train**: It was assumed that people were commuting or travelling into Edinburgh from a distance of 30 km or less; for this line length the average train speed is about 60 km/h (17 m/s) ( Jiang *et al.*, 2024).
- **Car:** Traffic was monitored before and after 20 mph zones were introduced in Edinburgh.

The city centre speed after implementation was measured as 22 mph (10 m/s) ( Nightingale *et al.*, 2021)

- **Bus:** Assumed to be the same speed as a car. Although they stop more frequently (i.e., slower speeds), they also have access to bus lanes (i.e., faster speeds during periods of high traffic)
- **Van**: Assumed to be the same speed as a car
- **Cycling:** 12–13.9 mph, leisure, moderate effort, 13 mph used as mid-point of moderate effort: 13 mph=5.8 m/s
- **Walking:** 2.8 to 3.4 mph, level, moderate pace, firm surface Mid-point=3.1 mph (1.4 m/s)

The following list presents carbon footprints per km for each transport mode. These estimates are based on Our World in Data (2023), unless otherwise stated.

- **Car (diesel):** 171 g
- **Car (petrol):** 170 g
- **Van**: 170 g
- **Car (non-plug in hybrid):** 119 g (midpoint between petrol and hybrid car)
- **Bus:** 97 g
- **Car (hybrid):** 68 g
- **Walking:** 55 g
- **Car (electric):** 47 g
- **Cycling:** 35 g (midpoint from Leurent [2022])
- **Train**: 35 g

***Traffic displacement.*** The minimum distance from the full home postcode to the nearest segment of the LEZ boundary was calculated for each respondent using the open-source software, QGIS (v3.30.0) ( https://qgis.org). To examine any potential traffic displacement, taking account of the potential discrepancy between reporting of postcodes and living inside/outside the LEZ boundary, we compared two sets of respondents who reported “more traffic” or “less traffic” as a reason for any changes to frequency, duration, or mode in commute or other travel. The data for inside and outside commuters, as well as people who travel to the LEZ for other reasons, was used for this analysis.

One set of respondents included those who reported living inside or outside the LEZ, and the other set used postcodes to define living inside, ≤1 km outside the boundary, or >1 km outside the boundary. The latter set excluded any respondents who reported living inside the LEZ, but did not have an outcode within the LEZ (i.e., EH1, EH2, EH3, or EH8).

## Results

Both surveys were available from 4 ^th^ October, with the panel survey remaining open until 14 ^th^ October and the community version closing on 15 ^th^ November 2024. A total of 351 respondents completed the panel survey and a further 260 submitted the community version (i.e., total *n*=611). Thirty-four respondents were disqualified from the panel survey and eight from the community survey for answering “No” to the question, “Do you agree to provide feedback about Edinburgh’s Low Emission Zone?”, or not commuting or travelling to the LEZ for other reasons. There were similar proportions of males (167; 48%) and females (179; 51%) in the panel version, with proportionally slightly more male respondents (145; 56%) in the community survey. There was a greater number of respondents aged 24–44 years in the panel version, with fewer respondents in the youngest and oldest age brackets in the community survey (see Table 1). In both surveys, and more prominently in the community results, the mode SIMD was decile 10 (i.e., least deprived) (see Table 1).

**Table 1.** Descriptive statistics (n [%]) of the demographic characteristics in the panel and community surveys.

Demographic characteristic	Survey	
*Age*	Panel	Community
18-24	37 [11%]	9 [3%]
25-34	79 [23%]	48 [18%]
35-44	72 [21%]	59 [23%]
45-54	49 [14%]	59 [23%]
55-64	52 [15%]	58 [22%]
65+	59 [17%]	22 [8%]
Prefer not to say	3 [1%]	5 [2%]
*SIMD 2020 Decile*		
1	8 [2%]	1 [<1%]
2	19 [5%]	7 [3%]
3	30 [9%]	15 [6%]
4	43 [12%]	17 [7%]
5	34 [10%]	24 [9%]
6	29 [8%]	16 [6%]
7	35 [10%]	35 [13%]
8	38 [11%]	25 [10%]
9	38 [11%]	23 [9%]
10	69 [20%]	80 [31%]
Not found	6 [2%]	12 [5%]
Prefer not to say	2 [1%]	5 [2%]

Self-reported happiness and wellbeing were generally high in both the panel and community surveys, with 75% or more respondents scoring 6 or above on an 11-point scale with 10 being the highest. The response to the question, ‘How anxious did you feel yesterday?’, had a more uniform distribution across the 11-point scale for both the panel and community results.

### Travel behaviour inside and outside the Low Emission Zone (LEZ)

More respondents commuted inside the LEZ in the panel (n=178, 51%) and community (n=174, 67%) surveys than outside the LEZ boundary (panel n=89, 25%; community n=52, 20%). Table 2 presents current commuting frequency, mode, and duration separately by travel inside and outside the LEZ for both the panel and community surveys. Respondents who reported travelling to the LEZ for other reasons (panel n=83, 24%; community n=34, 13%) are presented separately in Table 3. The frequency of travel was different inside and outside the LEZ (panel only), and using a single transport mode was more common inside the boundary. Commute durations were higher inside the LEZ than outside, though with high variability ( Table 2).

**Table 2.** Descriptive statistics (n [%]) of current commuting habits inside and outside the Edinburgh Low Emission Zone (LEZ) reported in the panel ( *n=*267) and community ( *n=*226) surveys.

Commuting characteristics	Panel survey			Community survey		
	Inside	Outside	*p*-value	Inside	Outside	*p*-value
Frequency (weekly) <1 1 2 3 4 5 >5	10 [5.6] 15 [8.4] 34 [19.1] 34 [19.1] 18 [10.1] 49 [27.5] 18 [10.1]	18 [20.2] 3 [3.4] 15 [16.9] 12 [13.5] 11 [12.4] 28 [31.5] 2 [2.2]	0.002	10 [5.7] 21 [12.1] 21 [12.1] 49 [28.2] 20 [11.5] 40 [23.0] 13 [7.5]	3 [5.8] 5 [9.6] 5 [9.6] 9 [17.3] 4 [7.7] 22 [42.3] 4 [7.7]	0.220
Transport mode Single Multiple *	86 [48.3] 92 [51.7]	67 [75.3] 22 [24.7]	<0.001	96 [55.2] 78 [44.8]	37 [71.2] 15 [28.8]	0.020
Duration in minutes (median [IQR **]) Single Multiple *	38 [31] 66 [63]	22 [28] 44 [44]	0.017 0.691	38 [23] 91 [59]	22 [16] 67 [60]	0.529 0.340
Number of respondents	178 (51)	89 (25)	N/A	174 (67)	52 (20)	N/A

* May represent multiple journeys ** IQR=Interquartile range

**Table 3.** Descriptive statistics (n [%]) of other travel habits inside the Edinburgh Low Emission Zone (LEZ) reported in the panel and community surveys.

Other travel characteristics	Survey	
	Panel	Community
Frequency (weekly) <1 1 2 3 4 5 >5	42 [50.6] 16 [19.3] 9 [10.8] 9 [10.8] 5 [6.0] 1 [1.2] 1 [1.2]	9 [26.5] 4 [11.8] 4 [11.8] 7 [20.6] 4 [11.8] 1 [2.9] 5 [14.7]
Transport Mode Single Multiple	39 [47.0] 44 [53.0]	7 [20.6] 27 [79.4]
Duration (median [IQR *]) Single Multiple	22 [16] 63 [53]	22 [8] 60 [46]
Total	83	34

*IQR=Interquartile range

**Figure 2.**
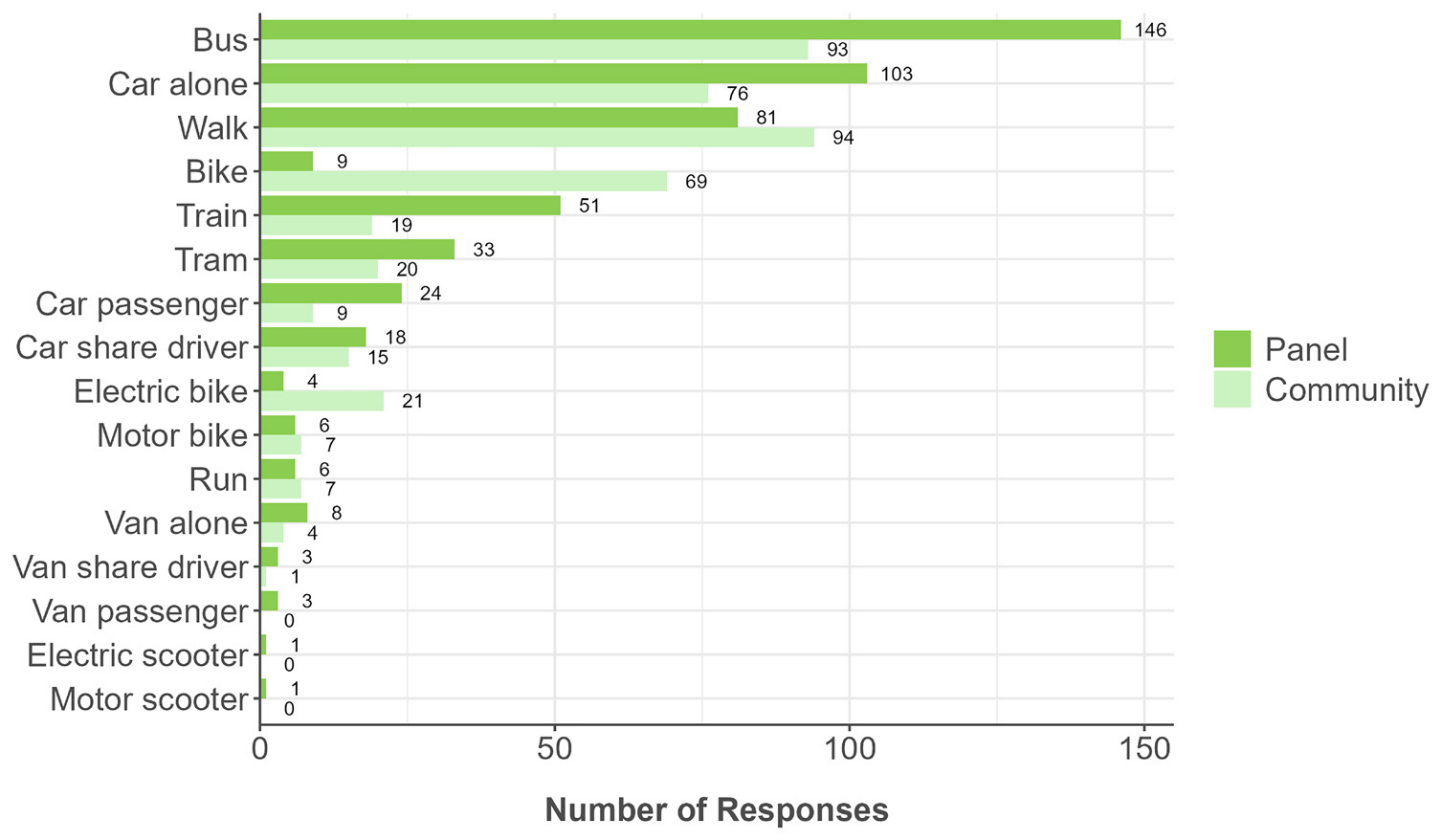
The transport modes reported in the **a**) panel ( *n*=267) and **b**) community ( *n*=226) surveys for commutes either inside or outside the LEZ.

The most common five modes of transport reported in the panel survey were bus, car (alone), walk, train, and tram. The corresponding list in the community survey was slightly different, including walk, bus, car (alone), bike, and electric bike (see Figure 2 for the full list of selected transport modes).

**Figure 3.**
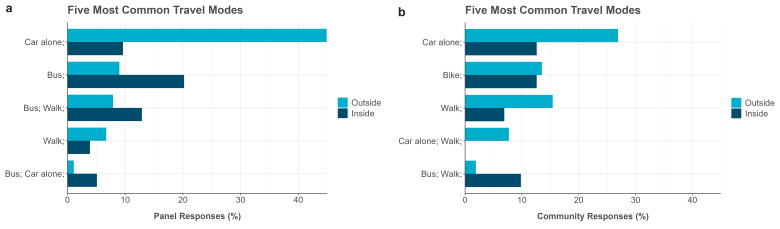
The most common reported modes of transport inside and outside the LEZ in the **a**) panel (inside LEZ, *n*=178; outside LEZ, *n*=89) and **b**) community surveys (inside LEZ, *n*=174; outside LEZ, *n*=52).

For the panel survey, there was a clear differentiation ( *p*<0.001) with more people using the car alone for their commute outside the LEZ. More people tended to use the bus inside the LEZ ( *p*=0.010). There were borderline significant differences in the proportions inside and outside the LEZ using bus & walk ( *p*=0.110) and walking ( *p*=0.054), but these comparisons were based on few respondents. There were similar findings in the community survey to that of the panel relating to use of the car, bus, and walking, with no difference in the proportion of people who cycle inside and outside the LEZ ( *p*=0.439) (see Figure 3). 

**Figure 4.**
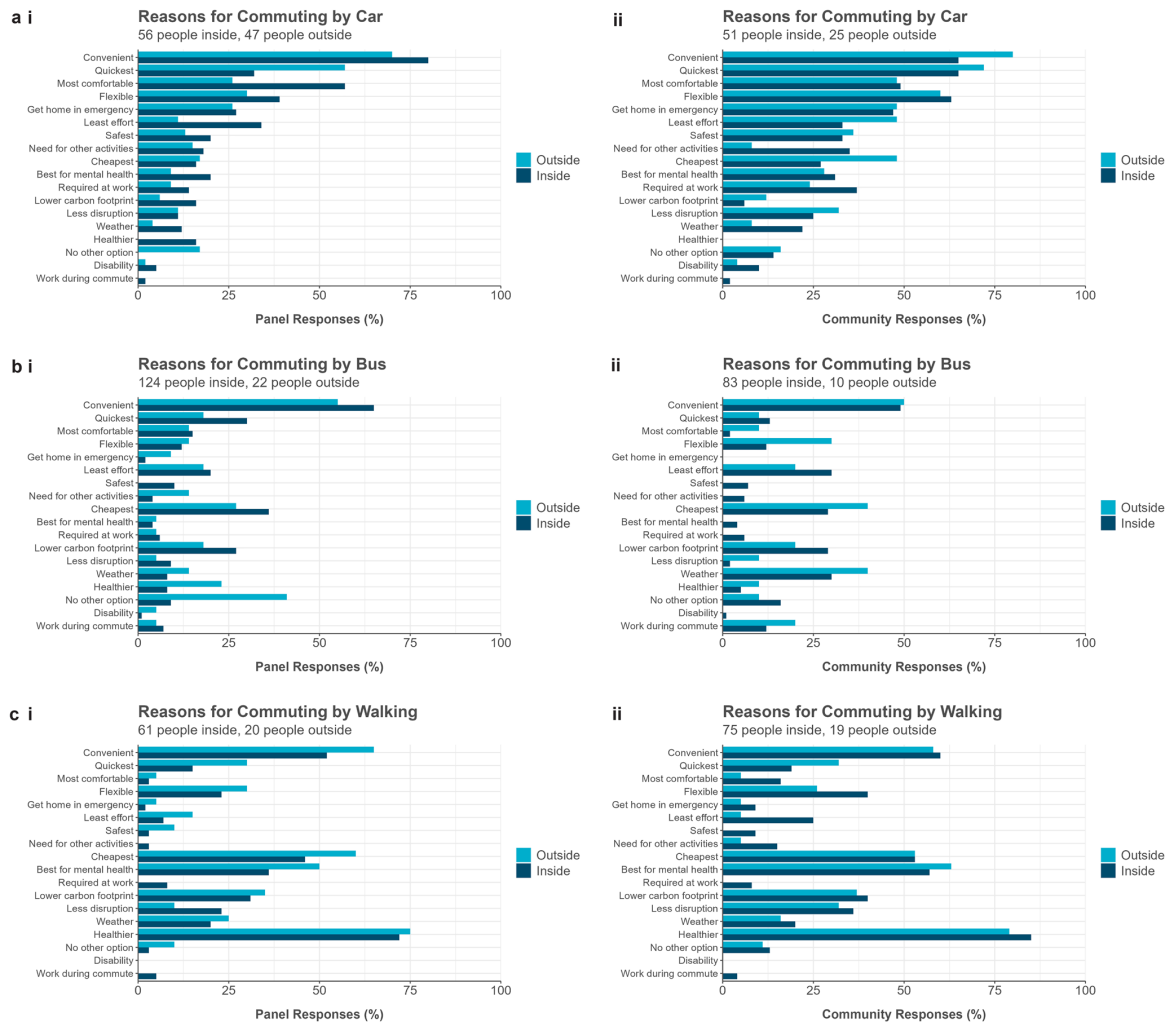
The percentage of **i**) panel and **ii**) community survey respondents reporting reasons for using **a**) car, **b**) bus, and **c**) walking by those commuting inside and outside the LEZ.

The reasons for using a given transport mode are depicted in Figure 4. For brevity, and since the three most common overall modes in the panel and community surveys were the same ( Figure 2), we present the individual reasons for commuting by car (alone), bus, and walking. As suggested by Figure 4, there are generally more differences between modes than either commuting inside or outside the LEZ or between the panel and community surveys. While convenience is clearly an important consideration for a given mode, ‘better for mental health’ and ‘healthier’ were much more common reasons for walking than car or bus.

### Travel behaviour before and after LEZ enforcement

Most respondents did not change whether they commuted inside or outside the LEZ following enforcement in June 2024, though more travel changes were observed for those commuting inside the LEZ (see Table 4). Of the 159 inside LEZ commuters in the panel survey, 70 (44%) reported a change in the frequency, duration, or mode of transport versus 8 out of 77 (10%) for the commuters outside of the LEZ. A similar trend was noted in the community survey, though to a lesser extent: 41 out of 171 (24%) of inside LEZ commuters reported such changes compared to 4 out of 47 (9%) of the outside commuters. The most common reason cited for a change in commute was “circumstance change”.

**Table 4.** The numbers (%) of respondents changing their commute from inside/outside the LEZ.

Commute		Survey	
Before enforcement	After enforcement	Panel	Community
Inside	Inside	159 (95)	171 (97)
	Outside	9 (5)	5 (3)
	Does not commute	0 (0)	0 (0)
Outside	Outside	77 (85)	47 (96)
	Inside	13 (14)	2 (4)
	Does not commute	1 (1)	0 (0)
Did not commute	Inside	6 (66)	1 (100)
	Outside	3 (33)	0 (0)

**Figure 5.**
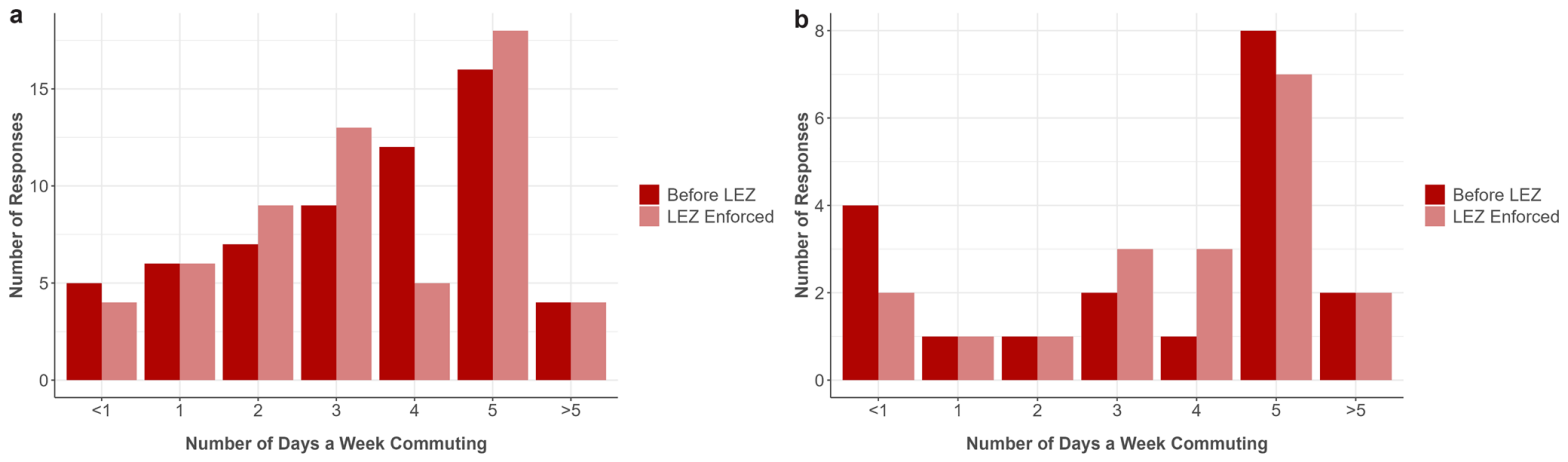
The changes to the commuting frequency in commuters travelling inside the LEZ before and after enforcement in the **a**) panel ( *n*=59) and **b**) community ( *n*=19) surveys.

**Figure 6.**
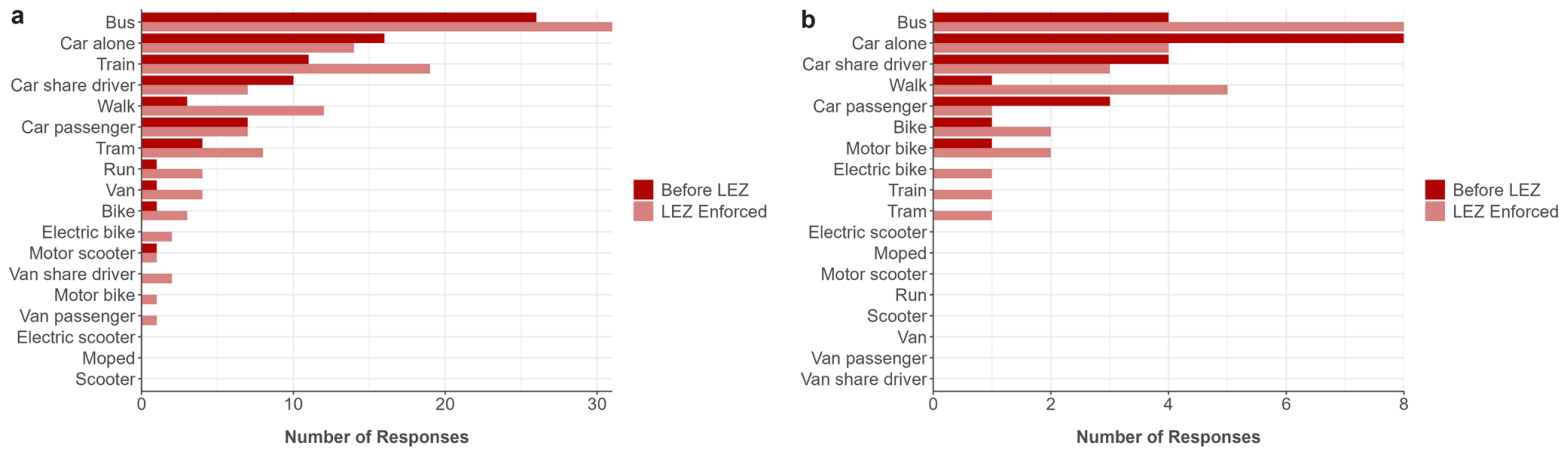
Reported commute modes for inside LEZ commuters before and after enforcement who changed mode of transport in the **a**) panel and **b**) community surveys (panel n = 45; community n = 10).

For the frequency of commuting, there was no general trend following LEZ enforcement in either the panel or community surveys ( Figure 5). However, the three most common reasons given in the panel survey (i.e., ‘quicker’, ‘more convenient’, ‘cheaper’) implied increasing frequency, whereas those in the community survey (i.e., ‘longer’, ‘less convenient’, ‘expensive’) suggested less frequent commuting ( Figure 6). A similar trend of disparate results between the panel and community surveys was also evident for changes in commute duration. The most reported reasons in the panel survey were ‘less traffic’, ‘public transport more reliable’, and ‘cycle routes improved’, indicating faster commute durations, in contrast to ‘more traffic’, ‘public transport less reliable’, and ‘different route’ (omitting ‘other’), as noted in the community survey ( Figure 6).

**Figure 7.**
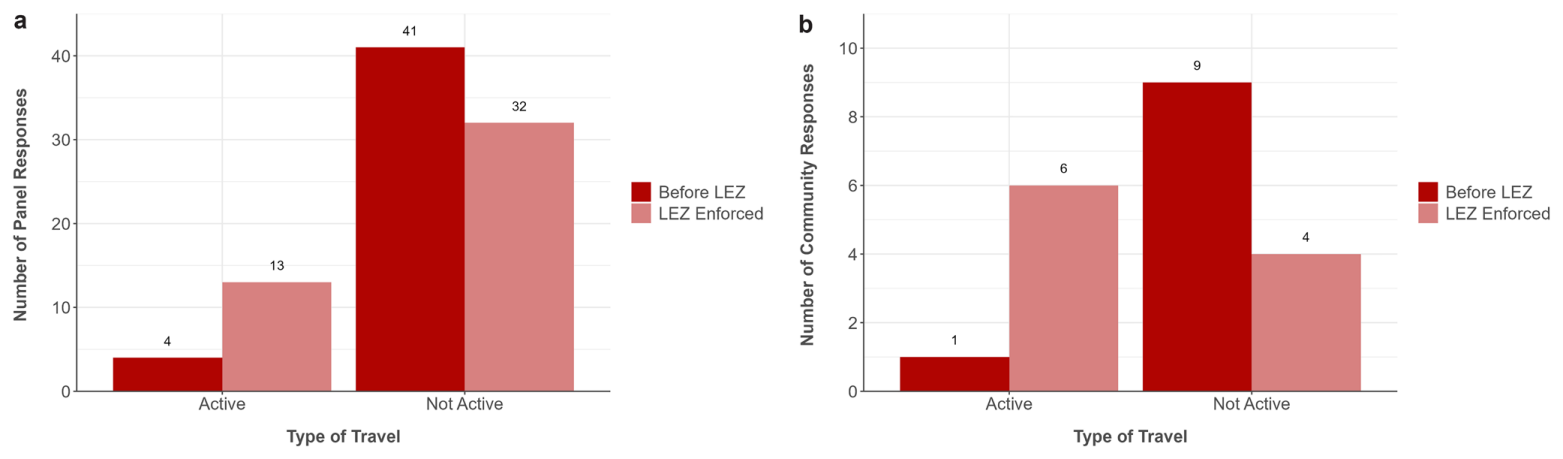
The number of participants who reported any changes to travel mode on the commute for those travelling inside the LEZ before and following enforcement in the **a**) panel ( *n*=45) and **b**) community ( *n*=10) surveys.

The shift in commute mode showed a general decline in the reporting of private vehicle use following the LEZ enforcement ( Figure 6). When categorising reported changes in commute modes for those commuting inside the LEZ before and after enforcement based on any active travel (i.e., including multiple modes), there was an increase in prevalence following enforcement in the panel ( *n*=53/178, 30% vs *n*=62/178, 35%) (Wilcoxon signed rank test *p*=0.014) and to a lesser extent in the community survey ( *n*=103/174, 59% vs *n*=108/174, 62%) ( Figure 7).

**Figure 8.**
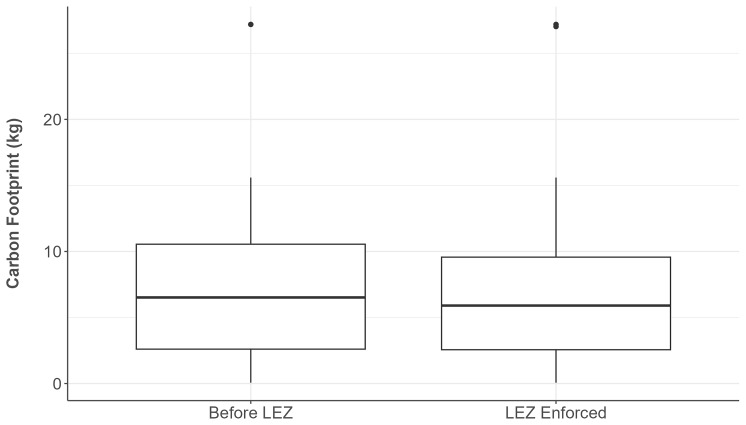
The carbon emissions estimated for those commuting inside the LEZ before and after enforcement for those using a single mode of transport ( *n*=58) in the panel survey.

The survey question regarding multiple commute modes contained up to 10 different reported modes of transport, suggesting respondents may have entered all possible modes of transport rather than only those used for a typical commute. Therefore, we limited the analysis of active travel minutes to respondents reporting a single travel mode (i.e., participants reporting multiple travel modes were excluded) ( Table 5). For insider commuters reporting a single mode, the median commute time was 22 minutes for both the panel and community survey (panel before [ *n*=6] and after [ *n*=5], community before [ *n*=34] and after [ *n*=34] LEZ enforcement). The weekly carbon emissions combined for respondents using a single mode prior to LEZ enforcement was 405 kg, which is only slightly higher than the combined emissions following enforcement (404 kg) ( *p*=0.798) (see Figure 8).

**Table 5.** The number of respondents using specific single mode travel for inside the LEZ before and after LEZ enforcement, as included in the carbon emissions analysis.

Mode	Before LEZ	LEZ Enforced
Bus;	28	28
Car alone;	20	17
Walk;	5	5
Car share driver;	2	2
Bike;	1	0
Car passenger;	1	1
Train;	1	3
Van;	0	2

### Impacts of the LEZ

**Figure 9.**
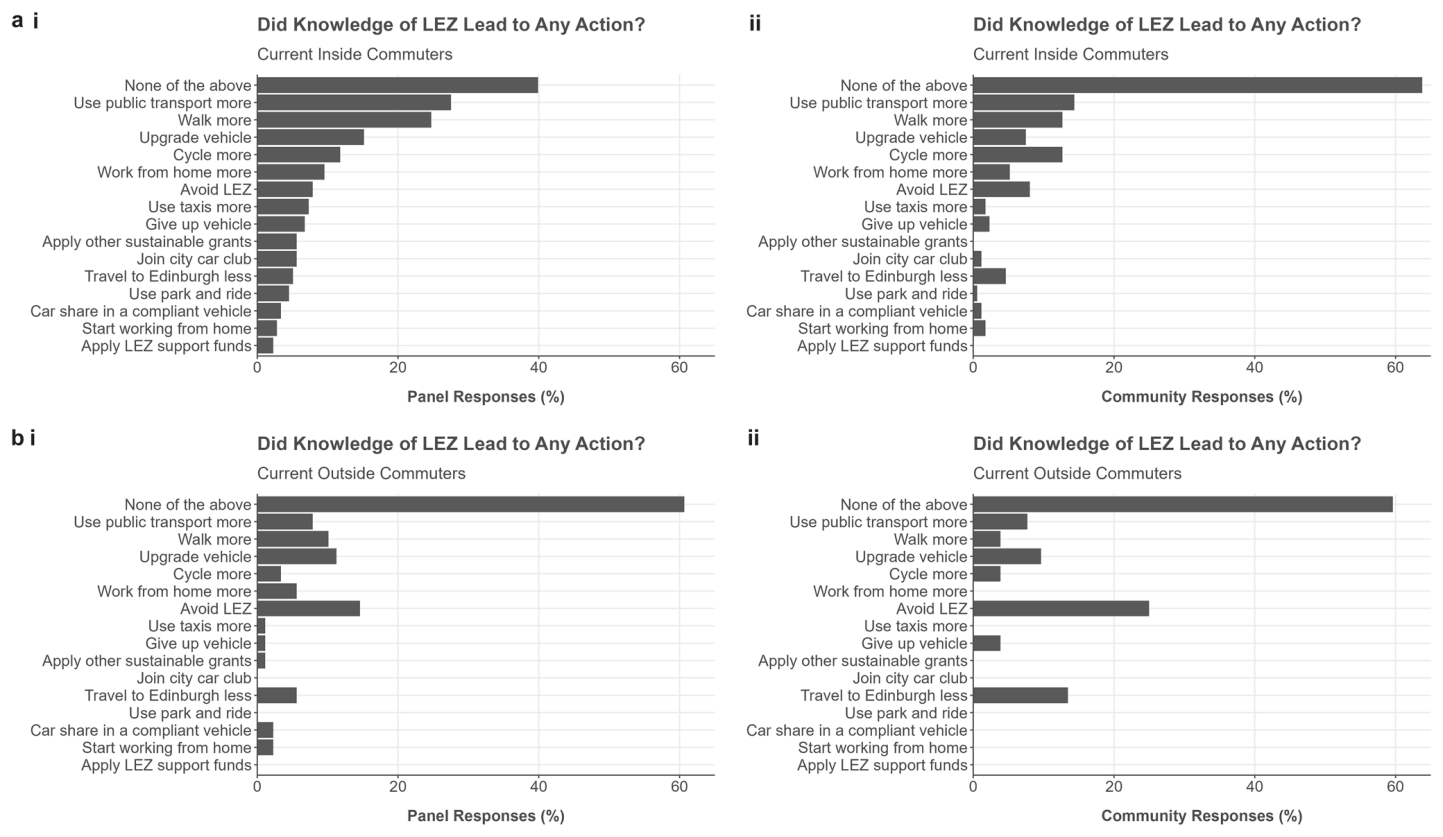
Reported actions taken as a result of knowledge of the LEZ in **a**) inside and **b**) outside commuters in the i) panel and ii) community surveys (inside: panel *n*=178, community *n*=174; outside: panel *n*=89, community *n*=52).

**Figure 10.**
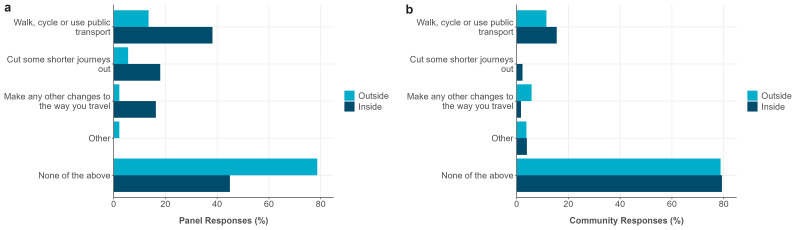
Reported actions for shorter trips (up to 5 miles) taken as a result of knowledge of the LEZ in inside and outside commuters in the **a**) panel (inside *n*=178, outside *n*=89) and **b**) community (inside *n*=174, outside *n*=52) surveys.

The survey asked participants explicitly about any changes prompted by their knowledge of the LEZ. Although the most frequent response was ‘none of the above’ for inside and outside LEZ commuters, more than half ( *n*=107, 60%) and over one third ( *n*=63, 36%) of inside commuters changed one or more travel behaviours in the panel and community surveys, respectively. Such behaviours included using public transport more and walking/cycling more. About 40% of outside LEZ commuters in each survey reported such changes (see Figure 9). For shorter journeys (defined as up to 5 miles), a greater proportion of those commuting inside the LEZ made changes to these journey types compared to those commuting outside the LEZ in the panel ( *n*=98/178, 55% vs *n*=19/89, 21%), but not community ( *n*=36/174, 21% vs *n*=11/52, 21%) survey (see Figure 10).

**Figure 11.**
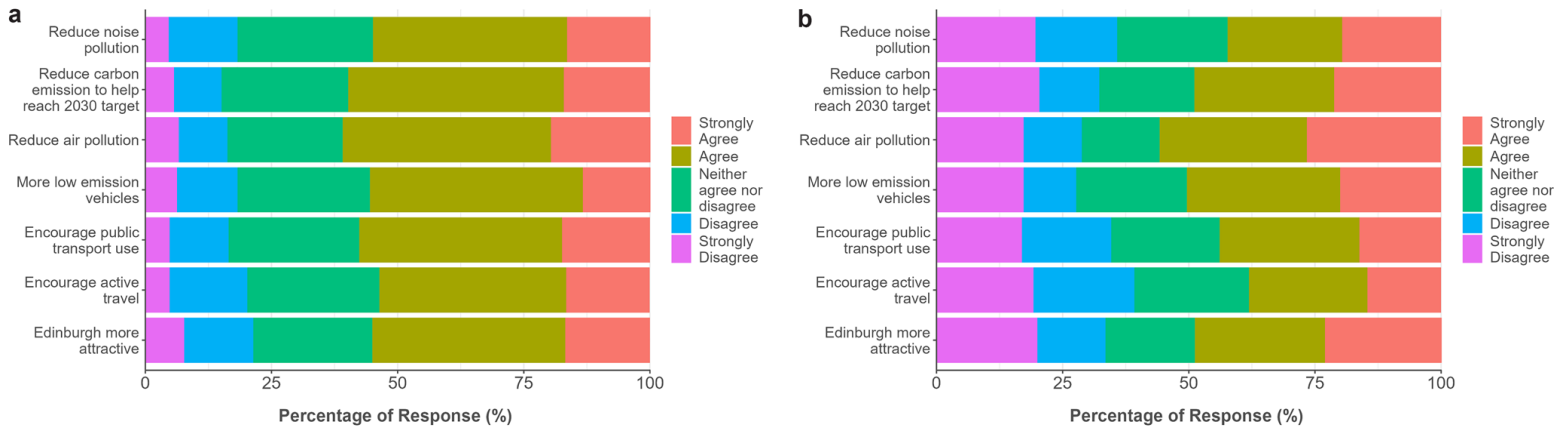
Agreement related to impacts of the LEZ for **a**) panel ( *n*=351) and **b**) community ( *n*=260) surveys.

The survey included questions on agreement related to seven specific impacts of the LEZ (e.g., reducing air and noise pollution). Responses to each of the individual impacts were broadly similar in the panel survey, with less than 25% disagreement and at least a 2:1 ratio of agreement to disagreement. The community survey suggested lower agreement overall and more variation across the different potential impacts; most agreement was indicated for ‘reduce air pollution’ and the most disagreement was for ‘encourage active travel’ ( Figure 11).

Respondents who did not commute for work or study reported other travel to the LEZ area (panel *n*=83, 24%; community *n*=34, 13%). The purpose of these journeys was predominantly for leisure or appointments, and the frequency was less often than that reported for commuters. Less than a third of these respondents (panel *n*=20, 24%; community *n*=10, 29%) reported changes (i.e., frequency, duration, mode) to their travel since enforcement. As observed in the results of those commuting into the LEZ, there was a general shift away from private vehicle use (though based on a smaller sample) (other travel results are presented in Table 3).

**Figure 12.**
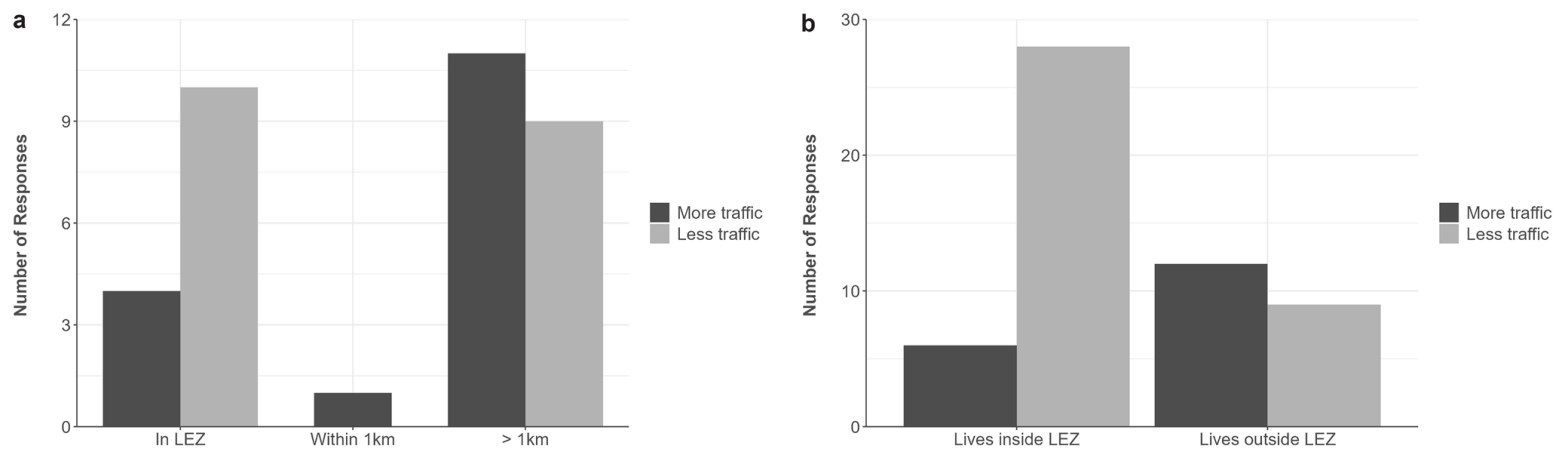
The traffic displacement analysis based on selecting “more traffic” or “less traffic” comparing **a**) reported postcodes ( *n*=35) and **b**) respondents who self-reported living within/outside the LEZ boundary ( *n*=55). Respondents who commuted or travelled for other reasons were included in the analysis (panel survey only).

There were 85 of the 351 (24%) respondents that reported living within the LEZ boundary. The main outcodes (i.e., first 3–4 digits of full postcode) within the LEZ are EH1, EH2, EH3 and EH8; 30 of the 85 reported living in one of these outcodes. Potential reasons for this discrepancy could be due to confusion about the specific location of the LEZ, having multiple home addresses, or reporting postcodes inaccurately. Overall, 55 people entered “More traffic” or “Less traffic” as a reason for a change to their commute/travel. Of these 55, 20 were excluded in the postcode analysis, as noted above. There did not appear to be evidence of more traffic outside the LEZ in either the postcode or self-reported data analysis (see Figure 12).

Free text comments were more prevalent in the community survey ( *n*=147/260, 57%) than submitted in the panel version ( *n*=128/351, 36%). The panel survey comments reflected more positive LEZ sentiments than those of the community respondents ( *n*=56/128, 44% vs *n*=35/147, 24%).

## Discussion

This study represents early results of travel behaviour and impacts of enforcement of the Edinburgh LEZ, with a focus on commuting for work or study. The most frequently reported commute modes overall were bus, car, and walking, with private car use more commonly reported for journeys that excluded the LEZ. Travel via the LEZ area was largely unchanged following enforcement, yet there were more changes in the frequency, duration, or mode in commutes that included the LEZ compared to those that did not. Initial results indicate more changes to shorter journeys for those commuting inside the LEZ, as well as a slight shift to more active travel. Nevertheless, some findings were mixed between respondents recruited by the two different methods.

It is perhaps not surprising that private car use was more common outside than inside the LEZ boundary. In general, city centres, where LEZs tend to exist, entail greater densities of services, more public transport options, and potential impediments to car use (e.g., higher cost/less availability of parking), thus facilitating other modes of transport ( Dempsey *et al.*, 2012). Nevertheless, other LEZs, such as Lisbon, have noted reductions in traffic of 3–4% both inside and outside the LEZ boundary, with a greater share of cars with higher emission standards ( Ferreira *et al.*, 2015). A 50% decrease in Euro 3 (and lower) vehicles was noted following the introduction of the Rotterdam LEZ, though these particular vehicles represented only 2% overall of those entering the LEZ area. This underscores that LEZs, depending on the specific parameters, may prevent only the most polluting vehicles and not necessarily traffic in general ( Attia *et al.,* 2023). Data available from Edinburgh Council suggests a decline in the number of non-compliant vehicles since enforcement began ( CEC, 2024b). An analysis of the Madrid LEZ found less traffic flow and greater public transport use into the LEZ, with some evidence of displacement (i.e., greater traffic levels) outside the boundary ( Tassinari, 2024). Our study specifically examined commuting to study/work, which may be different than travelling for other reasons, though we also collected this data from non-commuting respondents. Commuting behaviour may be distinct, as a study in China found individuals to be less likely to choose bus/walking during peak hours or when going to work ( Luan *et al.*, 2020). Such an effect, if relevant for the current study, would have led to an overestimate of overall car use to access the LEZ. 

LEZ enforcement did not appear to influence whether a commuting journey included the LEZ area. Significant commute changes are more likely to occur after moving home or changing employer ( Clark *et al.*, 2016). Nevertheless, there were more changes to the nature of the commute for those traversing the LEZ compared to those who did not. Following implementation of the Madrid LEZ, there was an observed 29 percentage point decline in private vehicle use, with 9 and 8 percentage point increases in public transport and active travel, respectively ( Tarriño-Ortiz *et al.*, 2022). Our results also indicated an increase in LEZ commutes with public transport and active travel, either as a component of or for the entire journey. These results coincide with a greater proportion reporting, as a result of the LEZ, walking, cycling or using public transport for shorter journeys inside compared to outside the LEZ, at least in the panel survey. Lower observed increases in active travel in the community survey could be due to a greater number of active travel users at baseline, as observed elsewhere ( Le Gouais *et al.*, 2021). Other studies have shown increases in public transport both inside and outside of the boundary, such as for the Rotterdam LEZ ( Attia *et al.*, 2023). However, such changes are not universal and may be context-specific: the Bradford Clean Air Zone, which charges larger vehicles (e.g., buses), but not private vehicles, found an overall increase in private transport to get to work ( Mebrahtu *et al.*, 2024). 

While the Edinburgh LEZ penalises cars below certain emission standards, the presence of an LEZ could raise awareness of environmental issues, such as air pollution and traffic, possibly leading to pro-environmental behaviour, including reduced car use ( PHS, 2023). Interestingly, in the case of the panel survey, there were similar levels of agreement across potential LEZ impacts, even for those which are less studied, such as noise reduction ( Andriolli & Silva, 2024). The free-text comments were more pro-LEZ in the panel compared to the community survey responses. With no comparable incentives, the community respondents’ stronger opinions about the LEZ or LEZ-related matters may have motivated their participation. Mebrahtu *et al.* (2024) also used two recruitment strategies (i.e., existing cohort and public samples) and found the public respondents to be more concerned about air quality issues.

There are numerous considerations for travel choices, including personal, socioeconomic, infrastructure and others ( Dingil *et al.*, 2021). A survey following LEZ implementation in Madrid found the most common reasons for transport choice were comfort, travel time, cost ( Tarriño-Ortiz *et al.*, 2022). These were also important motivations for travel mode in our survey, as well as the healthier option for non-motorised travel. 

### Strengths and limitations

Our study has several strengths. The survey represents short-term results of the travel behaviour impacts and perspectives from commuters on the enforcement of the Edinburgh LEZ. We collected detailed information involving travel inside and outside the LEZ, and before and following enforcement using two recruitment methods. To help with understanding the impacts, we included questions on specific actions attributed to knowledge/awareness of the LEZ. Several limitations should be noted with the interpretation of our results. Our survey used a cross-sectional approach and relied on the recall of individuals of travel behaviour prior to LEZ enforcement. While self-reported travel behaviour has been shown to be reliable at the group level ( Kelly *et al.*, 2014), recall of pre-enforcement travel may have been less accurate, particularly if the start of LEZ enforcement was not a particularly salient event ( Müggenburg, 2021). The use of objective data to assess traffic volumes or public transport use, for example, should be undertaken to compare with analyses based on subjective inputs ( Tassinari, 2024). Travel behaviour may have differed by season between the current (i.e., October/November) and pre-enforcement (i.e., pre-June) timelines. Some respondents may have been less likely to engage in active travel during potentially cooler and darker conditions as reported in the ‘current’ time-period, the impact of which may have downplayed any positive associations following LEZ enforcement ( Brainard *et al*., 2019). Impacts may have been underestimated, given the LEZ was implemented two years earlier (without issuing non-compliance fees); individuals may have undertaken specific changes prior to enforcement, which would not have been captured through the analysis of travel before and after enforcement. Nevertheless, we did ask about the replacement of non-compliant vehicles, which applied to a minority of respondents: four of eight individuals commuting inside the LEZ reported upgrading their non-compliant vehicle. Survey questions related to behaviour changes due to knowledge of the LEZ and agreement on potential LEZ impacts did not specify ‘enforcement’, so it was not possible to distinguish effects from implementations versus enforcement in those responses.

There were two notable issues with the collection of multi-modal data. First, it was apparent that the reporting of multiple modes may have represented separate commute journeys, which, if combined, would have overestimated commute duration. Second, the survey did not distinguish mode-specific duration of pre-enforcement travel. Thus, it is important for travel surveys to use explicit language for multi-modal travel to be able to answer the relevant research questions. There were inconsistencies between the locations of postcodes and whether a respondent reported living inside the LEZ. This weakened our ability to examine potential traffic displacement experienced by those living in close proximity to the LEZ boundary. Surveys were completed 4–5 months following the start of enforcement, so our results represent short-term findings, which should be compared with results over a longer follow-up period. The focus of our survey was on LEZ enforcement, though other concurrent policy/infrastructure changes may have influenced travel behaviour. For example, several respondents selected ‘cycle routes improved’ as the reason for changes in duration. The City of Edinburgh also increased parking fees ( CEC, 2024d) and introduced new cycle lanes in the city centre ( Walk Wheel Cycle Trust, 2024) only months before LEZ enforcement, potentially further discouraging private vehicle use. Subsequent research should examine LEZ enforcement impacts in the broader context of other mobility-related policies in Edinburgh and other urban settings.

In both the panel and community surveys, the proportions of respondents were mainly distributed across ages 25-64, though with lower proportions of 18-24-year olds compared to Edinburgh, particularly in the community cohort ( NRS, 2025). This demographic may have been less likely to receive/respond to online promotional content from the organisations involved in the study stakeholder group. More respondents were from the least deprived quintile according to the national SIMD rankings (panel: 31%; community: 40%), which is representative, but lower in fact, than the whole of Edinburgh (45%) ( CEC, 2020). Therefore, our findings may be less reflective of younger Edinburgh residents living in more deprived areas.

## Conclusion

We conducted a cross-sectional survey to understand the early impacts of the Edinburgh LEZ enforcement on travel behaviour and perspectives. We found some evidence that active travel and the use of public transport increased inside the LEZ boundary. Future research should confirm our findings with objective data and assess impacts over a longer time-period.

## Ethics and consent

Ethical approval for the study was granted by the Reading Independent Ethics Committee (RIEC) on 23 August 2024 (reference: 230824). Participants provided explicit written agreement to complete the survey before progressing to the questions.

## Data Availability

OSF: TRAVel Behaviour & Edinburgh Low Emission Zone. DOI: https://doi.org/10.17605/OSF.IO/AT8SV ( Mueller, 2025) This project contains the following underlying data: LEZ Travel Survey - Final.docx TRAVEL_opendata_v2.xlsx Data are available under the terms of the CC-By Attribution-NonCommercial-NoDerivatives 4.0 International

## Data statement

The TRAVEL study questionnaire and anonymised data are published on OSF ( http://doi.org/10.17605/OSF.IO/AT8SV).

## Information governance statement

*IOM is committed to handling all personal information in line with the UK Data Protection Act (2018) and the General Data Protection Regulation (EU GDPR) 2016/679. Under the Data Protection legislation, IOM is the Data Controller, and you can find out more about how we handle personal data, including how to exercise your individual rights and the contact details for our Data Protection Officer here.*
