# Supplementary material for: Travel behaviour and Edinburgh’s Low Emission Zone: a cross-sectional survey
Source: NIHR Open Res. 2026 Jan 19;5:33. Originally published 2025 Apr 14. [Version 2] doi: 10.3310/nihropenres.13891.2 (PMC13263679; doi:10.3310/nihropenres.13891.2)
Supplement: Peer review history

# Reviewer report: Reviewer response for version 2

**DOI:** 10.3310/nihropenres.15454.r39202

**Published:** 2026-06-12

**Authors:** Sean Beevers

**Affiliations:** 1 Imperial College London, London, UK

I looked at the author comments in response to my review and am happy that they have been answered.

Is the work clearly and accurately presented and does it cite the current literature?

Yes

If applicable, is the statistical analysis and its interpretation appropriate?

I cannot comment. A qualified statistician is required.

Are all the source data underlying the results available to ensure full reproducibility?

Yes

Is the study design appropriate and is the work technically sound?

Yes

Are the conclusions drawn adequately supported by the results?

Yes

Are sufficient details of methods and analysis provided to allow replication by others?

Yes

Reviewer Expertise:

I am an air pollution scientist and can provide general comments. I'd defer to a behavioural scientist/statististician for an assessment of the survey itself.

I confirm that I have read this submission and believe that I have an appropriate level of expertise to confirm that it is of an acceptable scientific standard.

# Reviewer report: Reviewer response for version 2

**DOI:** 10.3310/nihropenres.15454.r39203

**Published:** 2026-02-03

**Authors:** Rosemary McEachan

**Affiliations:** 1 Bradford Institute for Health Research, England, UK

Thank you for considering the reviewers comments and revising accordingly.

Is the work clearly and accurately presented and does it cite the current literature?

Yes

If applicable, is the statistical analysis and its interpretation appropriate?

Yes

Are all the source data underlying the results available to ensure full reproducibility?

Yes

Is the study design appropriate and is the work technically sound?

Yes

Are the conclusions drawn adequately supported by the results?

Yes

Are sufficient details of methods and analysis provided to allow replication by others?

Yes

Reviewer Expertise:

Complex intervention development and evaluation; evaluation of clean air zones; environmental determinants of health; cohort studies.

I confirm that I have read this submission and believe that I have an appropriate level of expertise to confirm that it is of an acceptable scientific standard.

# Reviewer report: Reviewer response for version 1

**DOI:** 10.3310/nihropenres.15098.r36351

**Published:** 2025-09-25

**Authors:** Sean Beevers

**Affiliations:** 1 Imperial College London, London, UK

Travel behaviour study Edinburgh LEZ

 This is a cross sectional survey looking at commuting behaviour inside and outside the Edinburgh LEZ. Key findings related to the mode usage, e.g. car, public transport inside and outside the LEZ zone with a small shift to active travel and public transport in the LEZ.

 I think this is a useful paper, looking beyond the air pollution impacts at travel behaviour. I’ve made some suggestions for the authors to consider which I hope are useful.

 Abstract:

 There needs to be a sentence on why the panel and community surveys.

 Also when it says “There was a clear differentiation in transport mode usage, with more people using the car alone for their commute outside the LEZ and more people using public transport inside the LEZ” make it clear whether this is prior to the LEZ or because of the LEZ.

 Main text:

 Although some of these points are addressed as limitations in the discussion I think they are important in interpreting the data, so further comment on these would improve the paper.

 A description of travel in the LEZ zone c.f. outside would be useful – prior to implementation, during the grace period and once implemented. Were there changes to other travel modes e.g. bus frequency, parking availability, no of cycle routes etc, which may have influenced the choices and change in modes reported.

 You also mention vehicle compliance, high for petrol cars, half for diesel, how did this change over time through the grace period inside the LEZ and around full implementation in 2024 and how did this compare with Edinburgh in general.

 Could the authors comment on how the survey accounts for the fact that the effects of the LEZ start during the grace period (start date in 2022). How would it have influenced the results.

 Also, the survey was 5 months after the 2024 start date which requires responders to think back to before LEZ. Was this in the grace period or maybe before that? Can you comment on the reliability of this data.

 The manuscript would benefit from more detail on the two survey’s - not only how representative they were of the wider population but also what were the reasons for having both community and panel results. Was it to compared strongly held opinions (community involvement) with more general opinions (panel)?

 Also, a statement on the degree to which the panel and community samples are comparable and support your results would be useful. For example: Fig 2 show differences in age….Fig 3 the panel and community are quite different, especially at the low SIMD deciles. Both have dominant decile 10. This seems unusual?

 It wasn’t clear to me why it was important to ask about “happiness yesterday” etc? I think this needs an explanation.

 How do you account for seasonal effects in your questions/data analysis. For example, if you survey in Oct/Nov for things like cycling now and compare this with what you did before LEZ (pre June 2024).

 It was confusing to use CO2 emissions as a metric and not include Nox emissions to assess the impact of the travel behaviour of the scheme? Was CO2 reduction a stated aim of the LEZ?

 In the commute modes analysis, I was also a bit surprised that bike/e-bike wasn’t chosen as it was relatively important and the differences between panel and community was large, which tells you something…your background talked a lot about active travel so it would seem to be a useful addition.

 Figure 8 – tiny numbers – are these results sound?

 Figure 12 – was CO2 emissions an aim of the LEZ…

 There are lots of figures – limiting the number may make your messages clearer.

Is the work clearly and accurately presented and does it cite the current literature?

Yes

If applicable, is the statistical analysis and its interpretation appropriate?

I cannot comment. A qualified statistician is required.

Are all the source data underlying the results available to ensure full reproducibility?

Yes

Is the study design appropriate and is the work technically sound?

Yes

Are the conclusions drawn adequately supported by the results?

Yes

Are sufficient details of methods and analysis provided to allow replication by others?

Yes

Reviewer Expertise:

I am an air pollution scientist and can provide general comments. I'd defer to a behavioural scientist/statististician for an assessment of the survey itself.

I confirm that I have read this submission and believe that I have an appropriate level of expertise to confirm that it is of an acceptable scientific standard, however I have significant reservations, as outlined above.

# Response

**Published:** 2025-12-04

**Authors:** Will Mueller

**Affiliations:** Institute of Occupational Medicine, Edinburgh, Scotland, UK

We thank the reviewer for their thoughtful and helpful input. We have responded below to the individual comments and hope we have sufficiently addressed all points raised. 

 I think this is a useful paper, looking beyond the air pollution impacts at travel behaviour. I’ve made some suggestions for the authors to consider which I hope are useful.

- **We thank the reviewer for this positive feedback.**

**Abstract:**

 There needs to be a sentence on why the panel and community surveys.

- **We have clarified that the two recruitment modes were to increase the number of survey responses.**

Also when it says “There was a clear differentiation in transport mode usage, with more people using the car alone for their commute outside the LEZ and more people using public transport inside the LEZ” make it clear whether this is prior to the LEZ or because of the LEZ.

- **We have confirmed that these results refer to the post-enforcement period.**

**Main text:**

 Although some of these points are addressed as limitations in the discussion I think they are important in interpreting the data, so further comment on these would improve the paper.

 A description of travel in the LEZ zone c.f. outside would be useful – prior to implementation, during the grace period and once implemented. Were there changes to other travel modes e.g. bus frequency, parking availability, no of cycle routes etc, which may have influenced the choices and change in modes reported.

- **We agree this would be useful contextual information to add. Although we did not have available information on all of the changes that took place across different travel modes during the different LEZ implementation phases, we have added the following sentence in the discussion to address this point: *The City of Edinburgh also increased parking fees (CEC, 2024d) and introduced new cycle lanes in the city centre (Walk Wheel Cycle Trust, 2024) only months before LEZ enforcement, potentially further discouraging private vehicle use.***

You also mention vehicle compliance, high for petrol cars, half for diesel, how did this change over time through the grace period inside the LEZ and around full implementation in 2024 and how did this compare with Edinburgh in general.

- **We only had this information for 2022 for the whole City of Edinburgh, based on a SEPA study. We have clarified this particular sentence in the methods: *Although 95% of petrol cars in Edinburgh were estimated to have been compliant at the time of LEZ implementation, only 50% of diesel cars met the required standard (CEC, 2023c).***

Could the authors comment on how the survey accounts for the fact that the effects of the LEZ start during the grace period (start date in 2022). How would it have influenced the results.

- **We agree this is an important point and had included the following excerpt in the discussion, noting the possible underestimation of LEZ impacts in our results:**
- ***Impacts may have been underestimated, given the LEZ was implemented two years earlier (without issuing non-compliance fees); individuals may have undertaken specific changes prior to enforcement, which would not have been captured through the analysis of travel before and after enforcement. Nevertheless, we did ask about the replacement of non-compliant vehicles, which applied to a minority of respondents: four of eight individuals commuting inside the LEZ reported upgrading their non-compliant vehicle. Survey questions related to behaviour changes due to knowledge of the LEZ and agreement on potential LEZ impacts did not specify ‘enforcement’, so it was not possible to distinguish effects from implementations versus enforcement in those responses.***

Also, the survey was 5 months after the 2024 start date which requires responders to think back to before LEZ. Was this in the grace period or maybe before that? Can you comment on the reliability of this data.

- **We thank the reviewer for noting this point and also considered this in the discussion with the below sentence. The actual survey wording was intentionally non-specific and referred to ‘before the LEZ enforcement,’ implying a comparison in the months before (i.e., during the grace period and not prior to implementation) and following enforcement (given the survey timing of 4-5 months afterward).**
- ***While self-reported travel behaviour has been shown to be reliable at the group level (Kelly et al., 2014), recall of pre-enforcement travel may have been less accurate, particularly if the start of LEZ enforcement was not a particularly salient event (Müggenburg, 2021).  ***

The manuscript would benefit from more detail on the two survey’s - not only how representative they were of the wider population but also what were the reasons for having both community and panel results. Was it to compared strongly held opinions (community involvement) with more general opinions (panel)?

- **We have noted in the methods that the two versions of the survey, based on different recruitment mechanisms, was to maximise the number of responses.**

Also, a statement on the degree to which the panel and community samples are comparable and support your results would be useful. For example: Fig 2 show differences in age….Fig 3 the panel and community are quite different, especially at the low SIMD deciles. Both have dominant decile 10. This seems unusual?

- **We have added in the strengths and limitations section a comparison of the study demographics in the panel and community surveys, in terms of age and deprivation (we did not collect ethnicity), compared to the larger Edinburgh population.**
- ***In both the panel and community surveys, the proportions of respondents were mainly distributed across ages 25-64, though with lower proportions of 18-24-year olds compared to Edinburgh, particularly in the community cohort (NRS, 2025). This demographic may have been less likely to receive/respond to online promotional content from the organisations involved in the study stakeholder group. More respondents were from the least deprived quintile according to the national SIMD rankings (panel: 31%; community: 40%), which is representative, but lower in fact, than the whole of Edinburgh (45%) (CEC, 2020).  Therefore, our findings may be less reflective of younger Edinburgh residents living in more deprived areas. ***

It wasn’t clear to me why it was important to ask about “happiness yesterday” etc? I think this needs an explanation.

- **The happiness and wellbeing questions were included to detect any differences between response subgroups and also to provide baseline information for potential future surveys.**

How do you account for seasonal effects in your questions/data analysis. For example, if you survey in Oct/Nov for things like cycling now and compare this with what you did before LEZ (pre June 2024).

- **This is an important point that we have now added to the discussion:**
- ***Travel behaviour may have differed by season between the current (i.e., October/November) and pre-enforcement (i.e., pre-June) timelines. Some respondents may have been less likely to engage in active travel during potentially cooler and darker conditions as reported in the ‘current’ time-period, the impact of which may have downplayed any positive associations following LEZ enforcement (Brainard et al., 2019).***
- **Brainard, J., Cooke, R., Lane, K. and Salter, C., 2019. Age, sex and other correlates with active travel walking and cycling in England: Analysis of responses to the Active Lives Survey 2016/17. *Preventive Medicine*, *123*, pp.225-231.**

It was confusing to use CO2 emissions as a metric and not include Nox emissions to assess the impact of the travel behaviour of the scheme? Was CO2 reduction a stated aim of the LEZ?

- **To clarify, we added the following underlined text in the methods section:**
- ***As an additional analysis, given the LEZ will contribute to Edinburgh’s 2030 Climate Strategy (CEC, 2021), we analysed the total commuting carbon emissions before and after enforcement for single modes of those commuting inside the LEZ.***
- **CEC, 2021. *2030 Climate Strategy – Delivering a net zero, climate ready city.* Last accessed 17 November 2025 from https://www.edinburgh.gov.uk/downloads/file/30783/2030-climate-strategy**

In the commute modes analysis, I was also a bit surprised that bike/e-bike wasn’t chosen as it was relatively important and the differences between panel and community was large, which tells you something…your background talked a lot about active travel so it would seem to be a useful addition.

- **We agree there were differences in the use of bike/e-bike in the community and panel surveys. In the inside/outside comparison, we examined the five most commonly reported travel modes, including those with multiple modes. ‘Bike’ was included in the community comparison, but not ‘e-bike’, the latter of which may have been reported alongside other modes such that it did not appear in the five most common. Including additional modes would have been a challenge to analyse with small samples sizes.**

Figure 8 – tiny numbers – are these results sound?

- **We have removed Figure 8 due to the small numbers involved.**

Figure 12 – was CO2 emissions an aim of the LEZ…

- **Please see our response three comments earlier.**

There are lots of figures – limiting the number may make your messages clearer.

- **We have combined the data from Figures 2 and 3 into what is now Table 1 and have replaced Figure 4 with text. Figure 8 and 16 have been removed.**

# Reviewer report: Reviewer response for version 1

**DOI:** 10.3310/nihropenres.15098.r35293

**Published:** 2025-06-03

**Authors:** Rosemary McEachan

**Affiliations:** 1 Bradford Institute for Health Research, England, UK

The authors report a cross sectional survey reporting changes in travel behaviour and perceptions amongst residents of a Scottish city following the enforcement of a low emission zone.

 The paper is well reported and comprehensive in methods, results and discussion.  The strengths and limitations are clearly articulated and appropriate.  I have made some suggestions which I feel would further improve the manuscript.

1. The introduction contains relevant material, but it would be good if the authors could clarify uncertainties and the novel contribution of the current study – what research gap is it trying to fill?
2. It would be useful if the authors could comment on the representativeness of both the community and panel sample in relation to general demographics of residents in Edinburgh (e.g. age, ethnicity, IMD indices). In the limitations section any major differences in sample characteristics should be noted, along with implications for interpretation.
3. The reporting is very comprehensive, but there are a lot of figures and tables, which can make it hard for the reader to find key material. For example could Figures 1-3 be combined into one table describing characteristics of the sample?
4. Some of the visualisations are reporting very small sample sizes (e.g. figure 8 – N=2; Figure 11, Figure 16). I’m not sure how useful these are, as a rule of thumb consider suppressing small sample sizes.

Is the work clearly and accurately presented and does it cite the current literature?

Yes

If applicable, is the statistical analysis and its interpretation appropriate?

Yes

Are all the source data underlying the results available to ensure full reproducibility?

Yes

Is the study design appropriate and is the work technically sound?

Yes

Are the conclusions drawn adequately supported by the results?

Yes

Are sufficient details of methods and analysis provided to allow replication by others?

Yes

Reviewer Expertise:

Complex intervention development and evaluation; evaluation of clean air zones; environmental determinants of health; cohort studies.

I confirm that I have read this submission and believe that I have an appropriate level of expertise to confirm that it is of an acceptable scientific standard, however I have significant reservations, as outlined above.

# Response

**Published:** 2025-12-04

**Authors:** Will Mueller

**Affiliations:** Institute of Occupational Medicine, Edinburgh, Scotland, UK

We thank the reviewer for their thoughtful and helpful input. We have responded below to the individual comments and hope we have sufficiently addressed all points raised.

 1.The introduction contains relevant material, but it would be good if the authors could clarify uncertainties and the novel contribution of the current study – what research gap is it trying to fill?

- **We have added to the background section that the study is to extend the limited evidence base on LEZs and active travel, which at present is mainly attributed to the London Ultra LEZ.**

2. It would be useful if the authors could comment on the representativeness of both the community and panel sample in relation to general demographics of residents in Edinburgh (e.g. age, ethnicity, IMD indices). In the limitations section any major differences in sample characteristics should be noted, along with implications for interpretation.

- **We have added in the strengths and limitations section a comparison of the study demographics, in terms of age and deprivation (we did not collect ethnicity), compared to the larger Edinburgh population.**

3.The reporting is very comprehensive, but there are a lot of figures and tables, which can make it hard for the reader to find key material. For example could Figures 1-3 be combined into one table describing characteristics of the sample?

- **We have combined the data from Figures 2 and 3 into what is now Table 1 and have replaced Figure 4 with text.**

4. Some of the visualisations are reporting very small sample sizes (e.g. figure 8 – N=2; Figure 11, Figure 16). I’m not sure how useful these are, as a rule of thumb consider suppressing small sample sizes.

- **We have removed Figures 8 and 16, which had small sample sizes. The others we have retained as they present useful results from our study. Some of the figures do show categories with fewer than 5 respondents, but we have included these as is for completion.**
